# The relationship between praying and life expectancy in cancerous patients

**Published:** 2015

**Authors:** N Hekmati Pour, H Hojjati

**Affiliations:** * Young Researchers and Elite Club, Aliabad Katoul Branch, Islamic Azad University, Aliabad Katoul, Iran; ** Department of Nursing, Aliabad Katoul Branch, Islamic Azad University, Aliabad Katoul, Iran

**Keywords:** prayer, life expectancy, cancer

## Abstract

**Introduction.** Knowing that someone was entangled with cancer is a surprising experience for that person. Being aware of having cancer not only makes the person loose his hopes and ambitions, but also influences his body and mental. Meanwhile, religion can play the proper role of complementary treatment, increasing life expectancy in these patients.

**Objective.** The study was conducted with the aim of determining the relationship between praying and life expectancy in cancerous patients.

**Method.** This descriptive correlation study was performed on 96 malignant patients who were under chemotherapy in Golestan province in 1392. Paloma and Pendleton’s Measure of Prayer Type questionnaires and Schneider questionnaire of life expectancy were used to collect this information. Analyses were performed by using SPSS 21.0. Data were analyzed by using the linear regression and the analytical significance was set at p < 0.05.

**Findings.** The linear regression showed a significant relationship between life expectancy and praying (CI95:0.01-0.13), OR = 0.07, Beta = -0.24 P < 0.02) and in the light of previous experience it showed a significant relationship between praying and life expectancy.

**Conclusion.** According to the obtained result of this study, cancerous patients can overcome their illness through praying, and they can also triumph cancer through self-confidence and control it, by getting more knowledge of their disease and become more hopeful about their future.

## Introduction

Cancer is a dangerous and horrible kind of illness that human has always suffered from that has also felt himself exposed to [**1**]. The awareness of having cancer is a surprising experience for everyone [**2**]. Recognizing it makes the person lose all his ambitions and hopes and affects not only his body but also his feelings [**3**]. Because of the nature of cancer, the patient has to accept the extended treatment through chemotherapy. Being frequently hospitalized also prevents him from having a normal life and the side effects of using chemotherapy takes the patient’s ability of enjoying his/ her life in different aspects [**4**]. The patient becomes tired, anxious, hopeless and sad when the treatment period prolongs [**5**,**6**]. The most hopelessness comes to the patients when they learn they suffer from malignant cancer [**7**]. In fact, in addition to the overwhelming harm of physical and functional problems that the cancerous patients encounter, they are faced with losing aim, worthlessness and meaningless, awkwardness and vanity of life [**8**]. At this time, hope is the best thing that can help the patients continue and develop their life [**4**]. Because hope is a significant factor in life that helps the cancerous patients overcome their problems and become compatible with their difficulties, making them able to reduce their torture and develop their quality of life [**2**]. 

It was confirmed that in advanced cancers, spirituality and religion have the primary role in life and life expectancy [**9**]. Spirituality can help the compatibility development of those people and their excitement flexibility through experience and positive feelings, considering the positive aspect of life and the enhancement of hope and satisfaction of life [**10**], because the people who believe in spirituality and use the spiritual methods to treat their illness have less mental disorders, pain, suffering, and anxiety [**11**]. Religion is known as a complementary method of treatment in medicine, and it seems that it can have a good effect when it is used together with some other treatment methods [**12**,**13**]. 

Among the spiritual and religious sources, the source that is used the most is praying [**14**], because the meaningful remembrance of praying and the ways that the existence of God and His connection to it is experienced is known as a rich source for the patients, when they are physically weak. When hospitalized, speculation enables him to tour to another place, and this place can be a caring place for him [**15**]. Believing in the mighty God and being in connection with Him who has domination over everything can make the people hopeful, can also promote their self-confidence, and prevents hopelessness [**16**]. Islamic psychologists believe that the remembrance of God is the way of mental disorder treatment such as anxiety and agitation and they also believe that people can stay away from the weakness by remembering God and having a connection with Him and reach the required relaxation and peace [**17**].

In the recent years, praying and the way of holding and performing it has been considerably growing with the help of the researchers. Smith (2013) believed that the praying treatment has a good result for the lung cancerous patients. Jahangeer (2009) believed that praying brought improvement of the hemoglobin impaired children. Sharifnia (2011) believed in the improvement of spiritual health by praying, Avazeh (2011) in the alleviation of burning pain, Etephagh (2009) in the carpal tunnel syndrome treatment, Tagheezadeh (2011) in the solemn gravity of the patients, Hariss (1999) in the heart disease [**15**,**18**-**23**].

It was also proved that praying has a good effect on diseases such as cardiovascular, AIDS, infertility, arthritis rheumatoid, thalassemia, prematurity, cerebrovascular accident [**19**]. Based on these aspects, patients suffering from chronic illnesses usually give up hope after some other treatments, or when they encounter these kinds of diseases, and these kinds of treatments are nothing but complimentary methods, the complimentary methods being those based on spirituality and used to combat hardship [**24**].

Religion has proven to be one method of treatment in complementary medicine [**9**,**25**]. Praying treatment is known in the world as a holistic treatment method [**19**]. However, unfortunately, patient’s religious needs are not considered in hospitals and it has frequently been observed that nothing related to the religion can be performed to improve the situations of the needy patients [**14**]. Researchers have become determined to do some studying based on the relationship between praying and the life expectancy of the cancerous patients because there are more than 70 million Muslims living in our country, who firmly believe in religion, and the religious value is combined with their lives [**15**].

## Method 

This descriptive correlational study was conducted on 96 cancerous patients who were under an agonist therapy treatment and who appealed to Food and Drug Laboratories Research Center of Golestan Medical University in the census in 1392. The inclusion criterion for this study was the recognition of cancer by an Iranian Muslim specialist. The exclusion criteria for the participants included all the patients who had the background of being hospitalized in a psychiatric ward and they had the experience of using substance drug abuses and hallucination substances such as amphetamine. If any of the participants did not like to take part in the study, they just did not fill in the questionnaire. 

The collecting tools of this study were the demographic surveys and a questionnaire that contained 32 questions related to Measure of Prayer Type, which was prepared by Poloma & Pendeltonin 1991 and then it was revised by Meraveglea in the same year [**26**]. This questionnaire was marked from 1 to 5 according to 7-Likert scale, and the range of the pray which was marked was of 32-224. The division of the pray was classified as weak (0-81), mean (81-162), high (163-244). This questionnaire was translated by Rezaee in Iran and then confirmed by Cronbach’s alpha coefficient, α = 0.79 being also confirmed. This questionnaire was used for the daily patients in 2009 and by Hojjati et al. in 2010 [**14**-**27**].

The third tool for this study was a questionnaire that contained 12 questions. These issues were Schneider questionnaire life expectancy which was marked by the 4-Likert scale, being were marked from 1 to 4, and the total target for life expectancy was 4-48, which was placed in three groups levels, the groups being classified in little (1-16), mean (17-32), and high (32-48). Moghimean (2011) calculated the reliability of this questionnaire by Cronbach’s alpha coefficient and α = 0.89 [**4**]. Before the beginning of the validity study, two surveys of life expectancy and frequency of praying were confirmed by ten faculties of Golestan Medical Science University and Open University of Katool.

For noble reasons, the approvals of the participants were not mentioned to ensure that their information is kept private. 

Normality was examined by a Kolmogorov-Smirnov test. Predictors were determined by using univariate and multivariate linear regression. The significance level was set at p < 0.05.

## Results

The typical age expanse of the participants was 57.5 ± 13, of whom 59% (55) were man and 41% (38) were women, 84% (87) of them were married, 16% (15) were single, 32.4% (30) were housewives, the residence of 61% (57 people) was city, 70% (65 people) had the experience of surgery in treating their cancer and 67.7% (63 people) had experienced chemotherapy.

The amount of praying results related to the mean and the standard deviation was 173.6 ± 33.1 higher than the average number, the value of life expectancy related to the mean and the standard deviation being 34 ± 10. There was a significant relationship between life expectancy and praying, (CI 95%: 0.01-0.13), P < 0.02, Beta = 0.24, and OR = 0.07. According to the previous experience, a significant relationship was shown between praying and life expectancy (**Table 1**).

According to the multivariate linear regression, there was a significant relationship between life expectancy and chemotherapy (CI 95%: -8.68-0.2), P < 0.029, Beta = 0.2 and OR = 4.3.

**Table 1:** The relationship between life expectancy and agonist therapy among the cancerous patients under treatment

Life expectancy /praying frequency	CI 95%	Beta	OR	P-value
Praying frequency	-0.004 - 0.18	0.19	0.08	0.06
Praying experience	0.05 - 0.45	0.21	0.25	0.01
Attitude toward praying	-0.1 – 0.39	0.11	0.14	0.26
General attitude of praying	0.01 – 0.13	0.24	0.07	0.02

## Discussion 

The outcome of this research showed that the amount of praying was almost higher than the usual, and it also revealed that there was a significant relationship between praying and life expectancy. This result could be related to the culture of our people. The life expectancy went up as praying became more prevalent, this being because of living in a Muslim country where praying is a major factor for the people. Religion was used as a complementary method in the medical science, and it was usually used as a mechanism against problems [**25**-**28**].

The people who believe in spirituality and use the spiritual method to treat themselves are less entangled with stress, anxiety, and mind disorders and, because they accept the existence of their illness, they can become more tolerant and more compatible [**11**].

Aghajanee (2011) showed in his study that 96% of the patients have a firm and dominant religious belief [29]. Hojateeet et al. showed a higher rate of using prayer, the mean of using prayer being of 174.4 ± 9.8 for the homo dialysis patients in Golestan province, which had the larger amount. Hojatee et al. (1388) also showed in a similar experiment in Amol that prayer has a higher rate than the usual mean (172 ± 10), also revealing that that experiment was in harmony with the present analysis [**14**,**27**].

Religious positive beliefs are important, and a useful factor in preserving health, at the same time being a good way of making the patient compatible with the illness. The moral actions such as praying make the patient be calm and have the effective compatibility against the disease, therefore, the patient will have less pain and the life expectancy will go up [**30**,**31**].

American magazines have expressed a relationship between spirituality and its different aspects such as prayer treatment, having discussed the connection between praying and the enhancement of health [**17**]. When a person becomes physically weak and is hospitalized, his imagination can enable him to go from one place to another. This place can be a curing place for him, encouraging him to take part in praying ceremonies. King believes that religion and praying make the people have a positive attitude towards the world, so it can drift the persons towards the illness and make them have more resistance against that disease, thus increasing the life expectancy [**27**]. 

Smith believes that the praying treatment has a positive effect [**18**]. Spirituality can increase compatibility; it can also have an effect on the flexibility of these patients, making them have a confident approach towards life, therefore improving the life expectancy, them being happier and more satisfied with their lives [**10**].

The result of this study showed a significant relationship between praying and life expectancy; it was somehow shown that if any of these two dimensions were raised, the patients’ life expectancy would improve.

In 2011, Hojjati showed in his study that the highest rate (44.5%) belonged to the patients who continuously thanked God because of His blessings. Also, 42% were always asking for God’s help, according to the last praying experience - 81.2%, more people used praying 19.8%, related to this belief, most of the patients were hopeful that God will cure them [**2**,**7**]. In most instances, it has been shown that the religious experiences have been very effective in solving the patient’s problems, pains, self-control, helped them have more confidence, and increased their compatibilities [**32**].

In 2011, Aghajanee showed in his study that praying and appealing to praying made the cancerous patient become more religious and increased the patient’s mental health [**29**], because the cancerous patients use religious beliefs, therefore, they have less anxiety and have a better relationship with their families and their relatives [**4**]. 

The result of this research revealed no significant correlation between praying and the demographic variable, but in 2006, Rezaee et al. showed a significant relationship between praying, gender, age, marital status, and education [**3**], the older the patients being the more dependent on praying than the ones who had primary education, women, and singles. In a 2009 study, Hojatee et al. highlighted a significant relationship observed between praying, age, and education [**3**].The older the patients were and the less education they had, the more they used praying [**14**]. Allen et al. (2004) pointed to this issue too; mentioning that willingness and tendency towards religion and spirituality increased as the age went up, being a way in which the patient can be compatible to his sickness and death [**33**]. Spirituality and religious deeds have an important role in the compatibility with the stressful conditions related to the chronic illnesses in higher ages [**34**]. In Maravilla (2002) and Algeer (2005) study, praying was more prevalent among the patients who had less education than between the ones with higher degrees and education, because they would use praying as a complementary treatment [**26**,**35**].

Moshe Mean et al. (2012) stated that there is no significant relationship between age, gender, marriage, and education regarding life expectancy, but the result of this study showed that there was a significant relationship between life expectancy and chemotherapy so that chemotherapy reduced life expectancy four times [**4**]. In addition, using any chemotherapy medicine can result in some physical consequences and can also change the patient’s mood, bringing down the value of the quality of life related to health during the period of treatment. Signs such as fatigue, a disorder in imagination, mental problems, can be observed during the therapy, showing that patients had a decline in the quality of their life after four months of treatment [**36**]. Spiritual beliefs not only give the patient more understanding but also make him become more compatible and handle stress easier. In addition, the pain reduces, and he likes to be more in contact with the people, thus becoming more sociable; thus, the positive spiritual belief is an important and useful factor for the patient compatibility and keeping his health [**30**]. 

The ones who believe in spirituality use spiritual methods for their treatment and are less entangled with pain and anxiety. Therefore, their strength and compatibilities increase by the acceptance of their illness. It seems that using spiritual experiences frequently along with the medical treatment have an excellent treatment effect, so it is recommended to have them both, as they are complementary [**11**]. When the classical treatments fail or when they become intolerable because of the side effects, the complementary medicine is the best choice [**22**]. At the time of illness, religious beliefs become critical to the patients because no other treatments have effect, thus patients getting more religious [**27**]. Religion has a definite value in filling up the vacancy of life, and it can protect patients against stress and make them compatible at the time of facing hardships, giving meaning to their lives [**14**].

Patients expect doctors and the other authorities to respect their religious beliefs; they also expect them to pay attention to their spiritual needs [**4**]. It is suggested that more extended studies should be done based on educational and cultural fields especially at the time of hardship and acceptance of the overwhelming treatments, because little research has been conducted on the religious and medical methods of treatment in Iran. We can also point to the deep feeling of some patients who have no tendency of showing any cooperation. Therefore, the number of the subjects is not enough, and because of this, we suggest a higher range of issues were approached to have a better study. 

According to the nature of cancer illness and the crises that it causes, such as spiritual crises and losing all the future hopes, religious beliefs and religion belongings can be a real relief and can have an excellent effect on the future of the patients. Therefore, the patients can overcome their illnesses with the help of praying and of other religious actions, thus helping them gain more power and being able to control their illnesses, as they know a lot about their sickness therefore they can stand the pain and suffering and become hopeful to future.

**Acknowledgment**

The authors would like to thank all the patients and the vice challenger coworkers in meal medicine section for the support in realizing this study. The authors would also like to thank for the financial support that the Open University of Aliabad Kool offered them. The number of the project is 5/ 2753 January 27, 2014.
